# A Permanent but Not Transient Postoperative Deficit Has Impact on OS and PFS in IDHwt-Glioma Patients

**DOI:** 10.3390/jcm15155835

**Published:** 2026-07-26

**Authors:** Julia Klingenschmid, Matthias Demetz, Nadine Pichler, Lukas Hagen, Marlies Bauer, Claudius Thomé, Christian F. Freyschlag, Aleksandrs Krigers

**Affiliations:** Department of Neurosurgery, Medical University Innsbruck, 6020 Innsbruck, Austria; ju.klingenschmid@tirol-kliniken.at (J.K.); matthias.demetz@tirol-kliniken.at (M.D.); nadine.pichler@tirol-kliniken.at (N.P.); lukas.hagen@student.i-med.ac.at (L.H.); marlies.bauer@tirol-kliniken.at (M.B.); claudius.thome@i-med.ac.at (C.T.); christian.freyschlag@i-med.ac.at (C.F.F.)

**Keywords:** deficit, glioma, hemiparesis, IDH wild-type, survival

## Abstract

**Background/Objectives:** Neurological impairment after glioma resection is a common postoperative event, but many deficits improve during recovery. The present study investigated whether transient and persistent postoperative neurological deficits differ in their association with overall survival (OS) and progression-free survival (PFS). **Methods:** We retrospectively reviewed data from patients who underwent initial surgical treatment for glioma in our institution. Neurological function was evaluated using documented clinical examinations performed before surgery, in the immediate postoperative period, and during routine follow-up 3–6 months after the procedure. **Results:** The study cohort comprised 496 patients, of whom 44.4% were women. The mean age at surgery was 60 years (95% CI, 58–61), and the average follow-up period was 21 months (95% CI, 19–23). According to the 2021 WHO classification, 80.0% of tumors were grade 4, 11.1% were grade 3, and 7.3% were grade 2. IDH mutations were absent in 81% of cases. Median survival for the entire cohort was 34 months (95% CI, 30–38). Preoperative neurological deficits, including hemiparesis, were not significantly associated with OS. Postoperative deficits that were resolved by the follow-up assessment showed no relationship with either OS or PFS, irrespective of IDH mutation status. In contrast, neurological deficits that persisted at follow-up were independently associated with shorter OS and PFS among patients with IDH-wild-type gliomas (*p* < 0.001), whereas no significant association was observed in the IDH-mutant subgroup. Similarly, when new neurological deficits were present at follow-up, poorer OS and PFS were predicted only in patients with IDH-wild-type tumors (*p* < 0.001). **Conclusions:** Temporary neurological deterioration following glioma surgery does not appear to adversely influence overall or progression-free survival, regardless of IDH status. However, persistent postoperative deficits, as well as newly acquired deficits that remain evident during follow-up, are strong indicators of an unfavorable prognosis in patients with IDH-wild-type gliomas.

## 1. Introduction

The development of a neurological deficit following glioma surgery is known to worsen overall survival [1,2,3]. These deficits include motor weakness and sensory impairment as well as disturbance of speech and vision and occur newly or worsened in around 30 to 40 percent [1,4,5] of patients after glioma surgery. Motor and speech deficits have the biggest negative impact on OS and PFS [1,3]. Usually, a postoperative neurological deficit appears or worsens directly after surgery without any delay and, if transient, most likely disappears within 3 months after surgery [4].

Independent factors such as extent of resection or surgery-associated ischemia and hemorrhage can influence the patients’ neurological status after glioma resection: surgery including resection of the supplementary motor area (SMA) has been associated with mostly transient contralateral weakness, apraxia, sensory disturbance or aphasia and even more so if the cingulate gyrus was included in the resection. Recovery time in these transient deficits associated with SMA is often shorter than one month [6]. Postoperative MRI scans have shown ischemic areas in patients who developed new neurological deficits following glioma surgery, particularly when the central arteries (anteromedial, anterolateral or posterolateral central artery), temporal lobe, insula, operculum or deep perforating arteries were involved in the tumor [5,7,8,9,10]. Further, a postoperative hemorrhage in the tumor site can lead to clinical neurological deterioration, sometimes leading to reoperation [11].

Postoperative neurological deterioration remains one of the major concerns in glioma surgery. Previous studies have demonstrated that new or worsened neurological deficits after glioma resection are associated with reduced survival, reduced functional independence, impaired quality of life, and limited access to adjuvant treatment [3,12,13]. The timing and dosing of adjuvant radiotherapy, for example, very much depend on Karnofsky Performance Status (KPS) and frailty rather than other factors such as age and residual tumor volume, according to the current guidelines [14,15].

This study aims to establish the impact of transient deficits compared to permanent deficits on patients’ PFS and OS following glioma surgery and its relation to IDH mutation status.

## 2. Materials and Methods

In our institutional neuro-oncological database we collected data from patients who received glioma surgery in an 8-year period. Indications and decisions regarding tumor surgery were taken from the interdisciplinary tumor board conference. This study includes surgical patients at their first intervention only. The patient enrollment period was from 1 August 2024 to 1 November 2024.

According to patients’ clinical charts we retrospectively assessed their clinical neurological status before surgery, before discharge and in a routine follow-up visit three to six months after surgery. Functional status was routinely assessed at these chronological moments by determining KPS and Rockwood’s Clinical Frailty Scale (CFS) [16]. Additionally, we gathered epidemiological and neuropathological data as well as imaging reports of pre- and postoperative magnetic resonance imaging (MRI) scans. Based on the time of diagnosis, the revised 4th or 5th WHO classification of central nervous system tumors was used to assess the respective histological diagnosis [17,18]. Immunohistochemistry (IHC) was used to evaluate the R132H IDH1 mutation and, in case of a negative mutation status, DNA sequencing was conducted for patients under 40 years to confirm IDH-wild-type status.

For statistical analysis, IBM SPSS Statistics (IBM SPSS Statistics for Mac OS, Version 27.0. Armonk, NY, USA: IBM Corp.) was used. *T*-tests were performed to determine scale variables, which are shown as means with standard deviations (SDs) due to normal distribution of the respective data. The chi-squared test was used to compare binominal pairs. The mean PFS and OS times were evaluated by performing Kaplan–Meier processing and the Log Rank test was added for the respective analysis. Analytical results with *p* < 0.05 were considered statistically significant.

## 3. Results

### 3.1. Demographics

A total of 496 patients (44.4% women and 55.6% men) were extracted from our institutional neuro-oncological database and included in this investigation. Mean follow-up of the total cohort was 21 months (CI 95% 19–23). Mean age was 59 years (CI 95% 58–61), mean KPI was 80 preoperatively and postoperatively, mean preoperative CFS was 3 and in the follow-up visit three to six months after surgery, mean KPI was 90 and mean CFS was 3. According to the WHO classification, 80% of the tumors (n = 397) were grade 4, 11.1% (n = 55) were grade 3 and 7.3% (n = 36) were grade 2 gliomas. A total of 98.2% did not receive radio- or chemotherapy prior to the surgery. Before their surgery, 97.3% showed some kind of neurological symptoms, 42.3% had hemiparesis and 30.8% suffered seizures prior to the surgery. Steroid therapy was administered in 67.6% preoperatively. Unilateral tumors were present in 88.6%; these were almost equally distributed with 51.1% in the left and 48.9% in the right hemisphere, and 1% of all tumors were located in the posterior fossa. Eloquent areas were affected in 45% of all gliomas.

Histologically, 81% were IDH-wild-type gliomas while 19% showed IDH mutation, MGMT promoter was methylated in 50.5%. While intraoperative navigation was used in all the cases, 5-ALA was administered in 51.6% and monitoring with intraoperative stimulation was used in 27.1%. In 19.4% only biopsy was conducted, 27.3% showed complete resection in the postoperative MRI scan and in 53.2% an incomplete resection was documented. A total of 74.1% of the patients were given postoperative steroid therapy. Adjuvant radio-chemotherapy was received by 67.5% of the patients, followed by additional chemotherapy in 50.4% of the cases—in 97% temozolomide was the agent of choice. A total of 14.4% received adjuvant therapy with tumor treating fields. Tumor progress at follow-up was found in 20.4%.

A tabular view of the demographic data is provided in Supplementary Table S1.

### 3.2. Neurologic Deficits

A focal neurologic deficit after surgery was found in 31.8% of the patients. At the follow-up visit, 45.9% presented with a permanent focal deficit. In 33.1% the postoperative deficit remained temporary and disappeared until the follow-up visit. A new deficit at follow-up was diagnosed in 16% of the patients.

### 3.3. Survival

#### 3.3.1. IDH Wild-Type (See Also Table 1 and Table 2)

Mean PFS was 15 months (CI 95% 13–18) and mean OS was 20 months (CI 95% 17–22) in patients with IDHwt gliomas. Patients presenting with hemiparesis at admission had a shorter PFS with 11 months (CI 95% 9–13) compared with 19 months in patients without hemiparesis (CI 95% 15–24, *p* < 0.01). Preoperative steroid therapy was associated with a reduced PFS of 13 months (CI 95% 11–16) versus 20 months in patients without steroids (CI 95% 15–25, *p* < 0.01), as well as a reduced OS of 17 months (CI 95% 15–20) versus 26 months (CI 95% 20–32, *p* < 0.01). Postoperative steroid therapy was associated with reduced OS and with a median survival of 16 months (CI 95% 14–18) compared with 32 months (CI 95% 25–40, *p* < 0.001) in patients who did not receive postoperative steroids. Eloquent tumor location was associated with a shorter PFS of 11 months (CI 95% 9–13) compared with 19 months (CI 95% 15–23, *p* < 0.001) for tumors in non-eloquent regions, and a shorter OS of 16 months (CI 95% 13–18) versus 22 months (CI 95% 19–26, *p* < 0.05). The presence of a permanent deficit at follow-up was associated with a PFS of 12 months (CI 95% 9–14) compared with 20 months (CI 95% 15–24, *p* < 0.001) in patients without permanent deficits and an OS of 20 months (CI 95% 17–23) versus 32 months (CI 95% 27–37, *p* < 0.001). Patients with newly developed deficits at follow-up had the poorest outcomes, with a PFS of 7 months (CI 95% 5–9) compared with 18 months (CI 95% 15–21, *p* < 0.001) in patients without new deficits and an OS of 14 months (CI 95% 11–17) versus 29 months (CI 95% 25–32, *p* < 0.001).

**Table 1:** Association of clinical characteristics and postoperative neurological deficits with PFS and OS in IDH-wt gliomas.

Variable	Group	PFS, Months (95% CI)	PFS *p*-Value	OS, Months (95% CI)	OS *p*-Value
Overall cohort (IDHwt)	All patients	15 (13–18)	–	20 (17–22)	–
Preoperative hemiparesis	Present	11 (9–13)	<0.01	NS	–
	Absent	19 (15–24)		NS	
Preoperative steroid therapy	Yes	13 (11–16)	<0.01	17 (15–20)	<0.01
	No	20 (15–25)		26 (20–32)	
Postoperative steroid therapy	Yes	NS	–	16 (14–18)	<0.001
	No	NS		32 (25–40)	
Eloquent tumor location	Eloquent	11 (9–13)	<0.001	16 (13–18)	<0.05
	Non-eloquent	19 (15–23)		22 (19–26)	
Permanent neurological deficit at follow-up	Present	12 (9–14)	<0.001	20 (17–23)	<0.001
	Absent	20 (15–24)		32 (27–37)	
New neurological deficit at follow-up	Present	7 (5–9)	<0.001	14 (11–17)	<0.001
	Absent	18 (15–21)		29 (25–32)	

NS = not significant, *p*-values according to Welch *t*-test.

**Table 2:** Kaplan–Meier curves show no significant reduction in OS and PFS in transient, but in permanent and new deficits at the follow-up visit in patients with IDHwt gliomas.

	IDHwt OS	IDHwt PFS
**Transient FND**	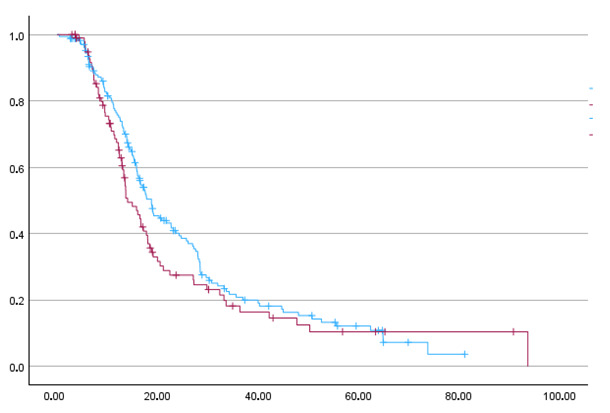	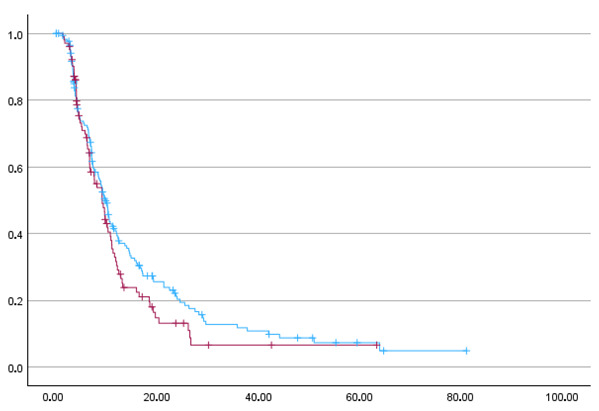
**Permanent FND at FU**	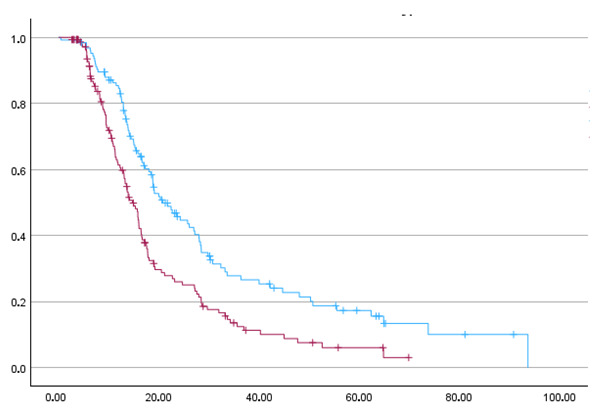	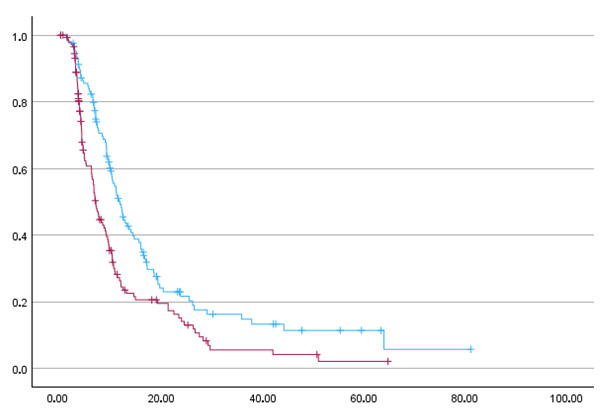
**New FND at FU**	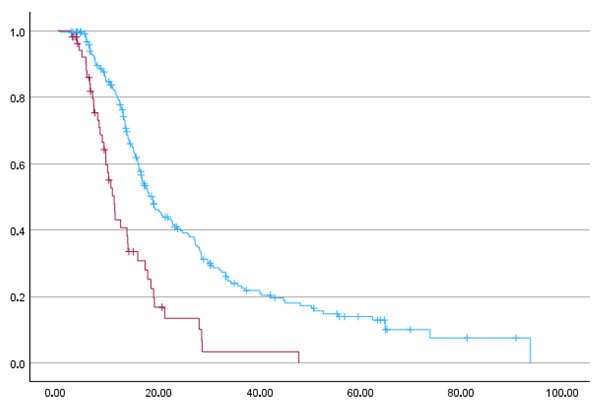	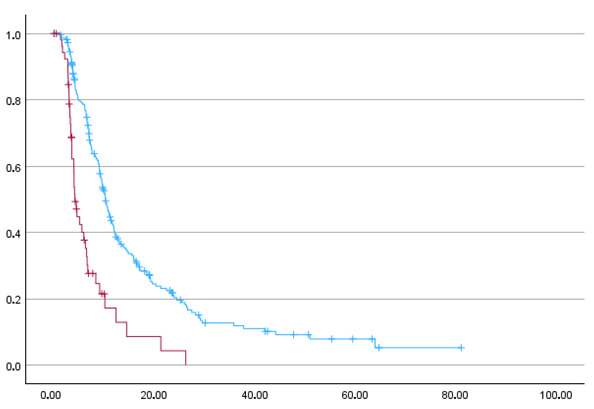

x-axes show the time to death or progression in months, y-axes show the cumulative survival, blue graphs indicate absence of respective deficits, red graphs indicate presence of respective deficits. FND = focal neurological deficit, FU = Follow up.

#### 3.3.2. IDH Mutation

Mean PFS was 66 months (CI 95% 57–76) and mean OS was 94 months (CI 95% 86–101) in patients with IDH-mutant gliomas (*p* < 0.001). In this subgroup, preoperative hemiparesis was not significantly associated with PFS. Similarly, preoperative steroid administration showed no significant impact on either PFS or OS. Postoperative steroid therapy was not associated with OS. Eloquent tumor location had no significant effect on PFS or OS in patients with IDH-mutant gliomas. Furthermore, neither permanent neurological deficits at follow-up nor newly developed deficits at follow-up were associated with reduced PFS or OS.

#### 3.3.3. Both IDHwt and IDH-Mutant Gliomas

Tumor location (left versus right hemisphere) did not significantly influence PFS or OS in either IDHwt or IDH-mutant gliomas. Postoperative steroid therapy was associated with reduced PFS in both molecular subgroups with a PFS of 25 months (CI 95% 20–30) compared with 37 months in patients not receiving steroid therapy (CI 95% 29–45, *p* < 0.001). Preoperative hemiparesis had no significant impact on OS. Whether a focal neurologic deficit was present or not immediately after surgery did not significantly affect PFS or OS, irrespective of IDH mutation status. A deficit that was present after surgery but disappeared until the follow-up visit had no relevance for PFS or OS in both IDHwt and IDH-mutant gliomas.

A simplified summary of the above-mentioned effects of neurologic deficits on OS and PFS is shown in Table 3.

## 4. Discussion

In this study, we investigated the prognostic relevance of transient and permanent postoperative neurological deficits after glioma surgery. Irrespective of IDH mutation status, transient deficits that resolved until the follow-up visit were not associated with impaired survival. Patients with IDH-wild-type gliomas showing permanent or new neurological deficits at follow-up had significantly shorter PFS and OS whereas in IDH-mutant gliomas neither permanent nor newly diagnosed neurological deficits at follow-up showed a significant impact on survival.

In line with previous reports [12,13], our data show that permanent neurological deficits are associated with worse outcome in patients with IDH-wild-type gliomas. We observed a particularly pronounced reduction in overall and progression-free survival when a new deficit was present at the follow-up visit. These findings support the concept that functional status is not solely a postoperative quality-of-life parameter, but more so a relevant outcome measure in neuro-oncological patients.

An important aspect of our results is the distinction between transient and permanent neurologic deficits. Functional decline immediately after surgery did not significantly influence PFS or OS, and deficits that resolved by the follow-up visit were not associated with inferior outcome. This observation is clinically relevant, as transient postoperative deficits are relatively common, especially following glioma resection involving eloquent or peri-eloquent brain areas. Rather than irreversible damage, these deficits can reflect temporary functional disturbance due to manipulation, edema, supplementary motor area involvement, or reversible metabolic or ischemic changes. Our findings suggest that, when followed by recovery within the early postoperative period, transient postoperative deficits do not entail negative prognostic outcomes.

In preoperative and intraoperative neurosurgical decision-making, this differentiation of transient and permanent deficits could become a relevant factor in select cases. While maximal safe resection remains a crucial goal in surgery of gliomas, the risk of a subsequent neurologic deterioration is always taken into consideration, as it affects the progress of rehabilitation and access to further oncological therapies. In tumors located in the supplementary motor area for example, a transient neurologic deficit may be acceptable under the circumstance that a meaningful tumor reduction is achieved and if a full functional recovery is expected. Nevertheless, a permanent neurologic deficit must be avoided particularly in IDHwt tumors where permanent deficits showed a strong association with inferior survival.

Several processes may explain the relationship between permanent deficits and worse survival in IDHwt gliomas. Firstly, current glioma treatment decision guidelines rely heavily on functional status, and the increased morbidity and mortality related to functional performance as represented by KPS and frailty has been shown in several studies [14,15,19,20,21]. Permanent deficits directly worsen performance status and therefore possibly cause limitations of the adjuvant therapy including aspects like feasibility, timing and intensity thereof [3]. Secondly, permanent deficits may be viewed as a result of aggressive tumor behavior and a more challenging resection technique. Patients harboring tumors involving eloquent regions, deep perforators, the insula, operculum, or central vascular territories are at increased risk for both incomplete resection and postoperative ischemic injury [10,22]. Lastly, a clinical neurological decline could also occur due to early tumor progression rather than surgical morbidity, especially when a new neurologic deficit is observed at the follow-up visit. In our patient cohort, PFS and OS were especially worsened when new deficits were detected at follow-up of IDHwt tumors, supporting the possibility that secondary neurological worsening could also be tumor progression-related.

Another notable finding in this study is the association between steroid therapy and poor outcome in IDHwt gliomas. Shorter PFS and OS were detected in both preoperative and postoperative steroid administration. However, this result may not necessarily be a direct causal effect of corticosteroids, as their use often results from mass effect, extensive edema and poor preoperative neurological condition. Nevertheless, the potentially biologically relevant effects of corticosteroids should not be neglected: immunosuppression due to steroids could interfere with antitumor immune responses and worsen the efficacy of emerging immunotherapeutic agents. Steroids have been shown to protect the tumor cells from genotoxic stress and therefore possibly imply a reduction in treatment effectiveness. Further, a steroid-related modulation of pathways in the tumor microenvironment has been postulated, and general side effects of long-term steroid use such as lower neutrophile count and hyperglycemia have also been taken into account [23,24,25]. Therefore, the clinical use of corticosteroids should remain as limited as possible as long as symptoms and edema can be adequately controlled.

This study showed no significant association between neurologic deficits and survival in IDH-mutant gliomas. This finding does not imply that functional deficits are unimportant in this patient group. Life expectancy is longer in patients with IDH-mutant gliomas [26,27,28] and a permanent deficit can profoundly affect these patients long-term. So even if there is not statistically measurable consequence regarding survival, the preservation of neurological function and a good quality of life is essential in this cohort. Possible reasons for the absence of a connection between IDH-mutant gliomas and permanent neurological deficits could be the longer natural history of the disease and the more favorable biological tumor behavior in the presence of IDH mutation.

Resulting from our data, preoperative hemiparesis was associated with worse PFS but not OS in IDHwt gliomas. Several factors, such as large tumor volume, involvement of motor tracts and edema can lead to hemiparesis before surgery. Its effect on PFS may be associated with limitations achieving maximal resection or with early tumor progression. Possible explanations for the absent influence on OS are adjuvant treatments, rehabilitation therapy, and the molecular variability in tumor biology.

This study has some limitations: Neurological status was assessed retrospectively from clinical documentation which may have introduced variability in the detection and grading of deficits. Further, during the eight-year period where patients have been included diagnostic classifications changed from the revised fourth to the fifth WHO classification which may have introduced some heterogeneity, even though tumors were classified according to the standard applicable at the time of diagnosis. Lastly, the smaller number of patients with IDH-mutant gliomas may limit subgroup analyses.

## 5. Conclusions

- Transient deficits resolving within three to six months after surgery do not impair survival in glioma patients, irrespective of IDH mutation status.
- Permanent and new neurological deficits at follow-up were associated with worse PFS and OS in IDH-wild-type gliomas, but not in IDH-mutant gliomas. Nevertheless, preserving neurological function remains essential in IDH-mutant patients due to their longer survival.
- This study emphasizes the importance of maximal safe resection with rigorous preservation of neurological function, particularly in IDH-wild-type gliomas.

## Figures and Tables

**Table 3:** The influence of neurologic deficits before surgery and at the follow-up visit including permanent, new and transient deficits on survival of patients with IDHwt and IDH-mutant gliomas.

Variable	IDHwt (81%)		IDHmut	
	**PFS**	**OS**	**PFS**	**OS**
Preoperative FND	↓	NS	NS	NS
Permanent FND at follow-up	↓	↓	NS	NS
New FND at follow-up	↓	↓	NS	NS
Transient FND (disappeared until follow-up)	NS	NS	NS	NS

Arrows indicate a decrease of survival. NS = not significant.

## Data Availability

The raw data supporting the conclusions of this article will be made available by the authors on request.

## Supplementary Materials

The following supporting information can be downloaded at: https://www.mdpi.com/article/10.3390/jcm15155835/s1. Supplementary Table S1 shows demographic details about the study population.

## Abbreviations

The following abbreviations are used in this manuscript:

OS	Overall survival
PFS	Progression-free survival
IDHwt	Isocitrate dehydrogenase wild-type
IDHmut	Isocitrate dehydrogenase mutated
SMA	Supplementary motor area
MRI	Magnetic resonance imaging
KPS	Karnofsky Performance Status
CFS	Clinical Frailty Scale
IHC	Immunohistochemistry
SD	Standard deviation
CI	Confidence interval
WHO	World Health Organization
NS	Not significant
FND	Focal neurological deficit
