# Supplementary material for: A Permanent but Not Transient Postoperative Deficit Has Impact on OS and PFS in IDHwt-Glioma Patients
Source: J Clin Med. 2026 Jul 26;15(15):5835. doi: 10.3390/jcm15155835 (PMC13466780; doi:10.3390/jcm15155835)
Supplement: Supplementary file 1 [file jcm-15-05835-s001.zip › jcm-4400608-supplementary.pdf]

Supplementary Table S1 shows demographic details about the study population.

Characteristic	Value
**Study population**	
Number of patients	496
Female sex	220 (44.4%)
Male sex	276 (55.6%)
Mean age, years (95% CI)	59 (58–61)
Mean follow-up, months (95% CI)	21 (19–23)
**Functional status**	
Mean preoperative KPI	80
Mean postoperative KPI	80
Mean follow-up KPI	90
Mean preoperative CFS	3
Mean follow-up CFS	3
**Tumor characteristics**	
WHO grade 2	36 (7.3%)
WHO grade 3	55 (11.1%)
WHO grade 4	397 (80.0%)
IDH wild-type	402 (81.0%)
IDH-mutant	94 (19.0%)
MGMT promoter methylated	250 (50.5%)
Unilateral tumor	439 (88.6%)
Left hemisphere	224 (51.1% of unilateral tumors)
Right hemisphere	215 (48.9% of unilateral tumors)
Posterior fossa location	5 (1.0%)
Eloquent location	223 (45.0%)
**Preoperative clinical characteristics**	
Any neurological symptoms	483 (97.3%)
Hemiparesis	210 (42.3%)
Seizures	153 (30.8%)
Preoperative steroid therapy	335 (67.6%)
Previous radio-/chemotherapy	9 (1.8%)
**Surgical characteristics**	
Intraoperative navigation	496 (100%)
5-ALA fluorescence	256 (51.6%)
Intraoperative stimulation/monitoring	134 (27.1%)
Biopsy only	96 (19.4%)
Complete resection	135 (27.3%)
Incomplete resection	264 (53.2%)

Characteristic	Value
**Postoperative treatment**	
Postoperative steroid therapy	368 (74.1%)
Adjuvant radiochemotherapy	335 (67.5%)
Additional chemotherapy	250 (50.4%)
Temozolomide as chemotherapy agent	243 (97.0% of chemotherapy-treated patients)
Tumor Treating Fields	71 (14.4%)
**Follow-up**	
Tumor progression at follow-up	101 (20.4%)
